# Quantitative analysis of annealing-induced instabilities of photo-leakage current and negative-bias-illumination-stress in a-InGaZnO thin-film transistors

**DOI:** 10.3762/bjnano.10.112

**Published:** 2019-05-27

**Authors:** Dapeng Wang, Mamoru Furuta

**Affiliations:** 1 Key Laboratory of Applied Surface and Colloid Chemistry, Ministry of Education; Shaanxi Key Laboratory for AdvancedEnergy Devices; Shaanxi Engineering Lab for Advanced Energy Technology, School of Materials Science and Engineering, Shaanxi Normal University, Xi’an 710119, China; 2 School of Environmental Science and Engineering, Kochi University of Technology, Kami, Kochi 782-8502, Japan; 3 Center for Nanotechnology in Research Institute, Kochi University of Technology, Kami, Kochi 782-8502, Japan

**Keywords:** metal oxide, photo-induced instabilities, photon energy, thermal annealing, thin-film transistor (TFT) device

## Abstract

This study examines the effect of the annealing temperature on the initial electrical characteristics and photo-induced instabilities of amorphous indium gallium zinc oxide (a-IGZO) thin-film transistors (TFTs). The extracted electrical parameters from transfer curves suggest that a low-temperature treatment maintains a high density of defects in the IGZO bulk, whereas high-temperature annealing causes a quality degradation of the adjacent interfaces. Light of short wavelengths below 460 nm induces defect generation in the forward measurement and the leakage current increases in the reverse measurement, especially for the low-temperature-annealed device. The hysteresis after negative-bias-illumination-stress (NBIS) is quantitatively investigated by using the double-scan mode and a positive gate pulse. Despite the abnormal transfer properties in the low-temperature-treated device, the excited holes are identically trapped at the front interface irrespective of treatment temperature. NBIS-induced critical instability occurs in the high-temperature-annealed TFT.

## Introduction

The rapid process of industrialization and commercialization has accelerated the development of consumption electronics and micromachining technology. One of the most successful modern-day microelectronic products are metal-oxide thin-film transistors (TFTs) that guarantee large-scale integrated circuits for applications in transparent and flexible flat-panel displays (FPDs). Amorphous InGaZnO (a-IGZO), an outstanding active channel material, is generally adopted in TFTs because of its high electron mobility, great environmental/thermal stability, and preparation versatility. The enhanced mobility of a-IGZO originates from the fact that the electrical conduction is insensitive to disorder because the conduction band edge depends on the In s-orbital [1]. However, there is a high density of oxygen-containing trap states (ca. 10^20^ cm^−3^) in the region above the valence band [2], which is sensitive to irradiation with light [3].

The typical staggered/coplanar TFT devices comprise several functional layers and their respective contact interfaces. Generally, the individual layer fabrication process follows a number of thin film deposition and photolithographic patterning steps. During the film growth through plasma-enhanced chemical vapor deposition or sputtering techniques, the sample suffers from the plasma bombardment in the chamber. In addition, the deposited films are inevitably irradiated by UV light in the process of patterning, inducing the increase in the concentration of oxygen-related defects, which further degrade the initial electrical properties and long-term stability. Therefore, many researches are devoted to reducing the density of defects during the fabrication, including the optimization of oxygen partial pressure [4], the increase of fabrication temperature, and the reduction of deposition power. In addition, post-annealing is recognized as an essential method to enhance the quality of the channel layer as well as its adjacent interfaces [5].

Although the initial performance of the TFTs can be improved using a passivation layer and suitable post-annealing, the devices in FPDs always undergo a negative gate bias and are exposed to the backlight for non-emissive displays. The degradation of oxide-based TFTs under this kind of negative-bias-illumination-stress (NBIS) is a key issue that has been investigated over the last decade [6–7]. Despite all efforts to unveil the mechanisms of NBIS, such as first-principles calculations and experimental researches, the NBIS-triggered degradations in devices annealed at different temperatures are still not fully understood. Furthermore, the photo-leakage current and NBIS-derived instabilities of devices annealed at different temperatures have not been quantitatively traced so far.

In this work, we investigate the impact of annealing temperature on the initial electrical characteristics and photo-induced instabilities in a-IGZO TFTs. The obtained electrical parameters imply that low- and high-temperature annealing leverage the generation of defects in the active layer as well as its adjacent interfaces. The incident photon energy (>2.7 eV) excites the defect generation and the leakage current increases in the devices regardless of heating temperature. By means of the double-scan mode and a positive gate pulse, the NBIS-caused hysteresis of the TFTs annealed at different temperatures is quantitatively monitored to trace trapped holes, generated donor-like states, and trapped electrons.

## Experimental

The bottom-gate top-contact a-IGZO TFTs are fabricated in a routine procedure [8]. The channel layer thickness is 45 nm, and then the TFTs are annealed at 300, 350, and 400 °C in N_2_ atmosphere for 1 h. The TFTs are designed with fixed width (*W*) of 50 μm and length (*L*) of 20 μm. The current–voltage (*I*–*V*) curves are measured using an Agilent 4156C semiconductor parameter analyzer at room temperature.

For the photo-leakage current measurements, monochromatic light with wavelengths λ of 400–530 nm is used. The details of the NBIS measurement (gate voltage *V*_GS_ = −20 V; λ = 460 nm) in double-scan mode are described in [8]. The duration of the NBIS measurements is 10^4^ s.

## Results and Discussion

**Figure 1:**
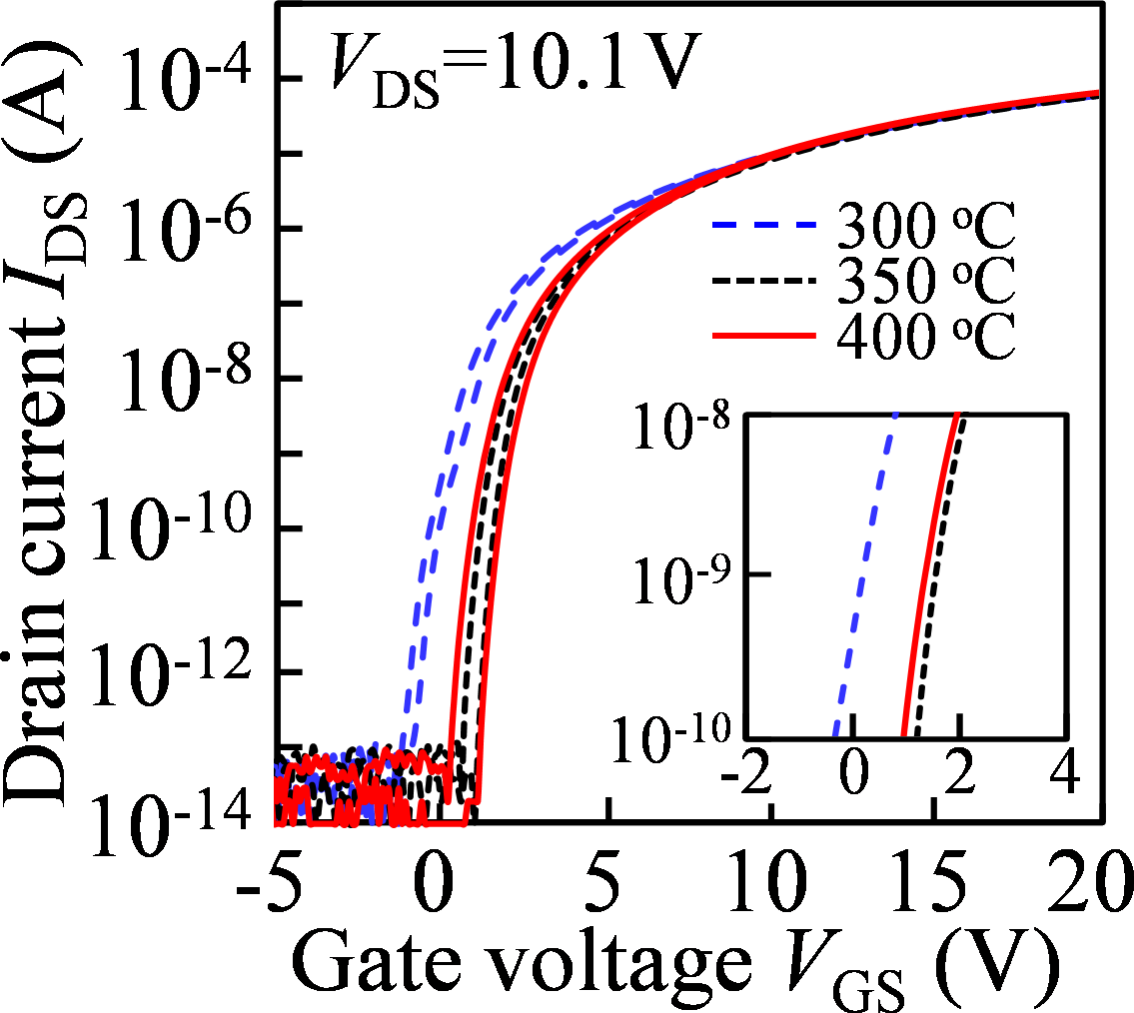
Transfer characteristics of the devices annealed at different temperatures.

Figure 1 displays the double-scan transfer characteristics (*V*_DS_ = 10.1 V) of the TFTs annealed at different temperatures. The extracted electrical parameters as functions of the treatment temperature are listed in Table 1. The field-effect mobility (μ_sat_) is directly proportional to the drain-to-source current (*I*_DS_) [9]. The mobility results demonstrate that the post-annealing temperature scarcely changes the *I*_DS_ values. The turn-on voltage *V*_on_ (at *I*_DS_ = 1 nA) of 0.17 V for the device treated at 300 °C changes to 1.53 and 1.33 V for the TFTs annealed at 350° and 400 °C, respectively. The shift of *V*_on_ in the positive direction suggests that a proper treatment temperature is beneficial for the formation of the metal-oxide atomic framework, improving the channel layer quality. The notorious *I*–*V* hysteresis is regarded as an indicator for the quality of the GI/semiconductor interface. The values of the hysteresis, i.e., the difference of *V*_GS_ at *I*_DS_ = 1 pA scanned in the forward and reverse directions, are 0.38, 0.37, and 0.77 V, for the devices annealed at 300, 350, and 400 °C, respectively. The large hysteresis implies that the high-temperature treatment causes the deterioration of the front interface. Furthermore, the subthreshold swing (SS = d*V*_GS_/dlog_10_(*I*_DS_)) is calculated to be 369, 250, and 292 mV/dec. for the TFTs treated at 300, 350, and 400 °C, respectively. Note that the SS is a measure for the total density of trap states (*N*_t_) in the semiconductor bulk and the front interface of a TFT device. On the basis of the functional relationship between of SS and *N*_t_ [10], an *N*_t_ value of 7.67 × 10^11^ cm^−2^·eV^−1^ is obtained for the TFT treated at 300 °C. From the comprehensive analysis of the hysteresis and SS results, we concluded that the low-temperature-annealed TFT exhibits the high density of defects in the IGZO bulk, whereas the high-temperature-treated device has severe defect states at the GI/IGZO interface. On the basis of our previous publication [11], the results suggest that the appropriate annealing temperature is helpful to suppress the defects and strengthen the quality of a-IGZO. However, the high temperature will induce a rupture of weak metal–oxygen bonds, introducing more defect sites.

**Table 1:** The electrical parameters of a-IGZO TFTs annealed at different temperatures.

	annealing temperature (°C)		
	300	350	400

μ_sat_ (cm^2^·V^−1^·s^−1^)	14.75	14.67	14.79
*V*_on_ at *I*_DS_ = 1 nA (V)	0.17	1.53	1.33
hysteresis Δ*V*_H_ (V)	0.38	0.37	0.77
subthreshold swing (mV/dec.)	369	250	292
total trap density (10^11^ cm^−2^)	7.67	4.72	5.76

**Figure 2:**
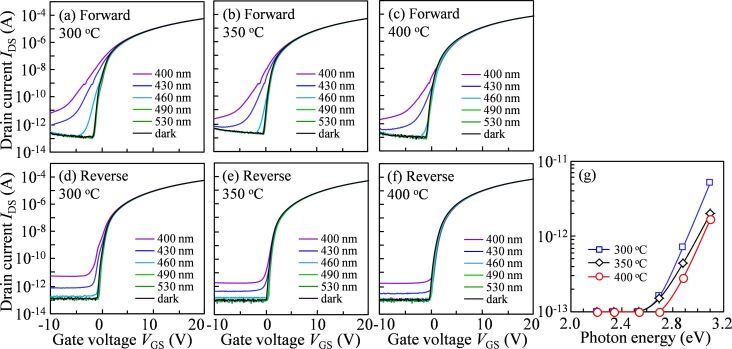
Transfer curves scanned in the forward direction for a-IGZO TFTs annealed at (a) 300 °C, (b) 350 °C, and (c) 400 °C and scanned in the reverse direction for the devices treated at (d) 300 °C, (e) 350 °C, and (f) 400 °C under monochromatic light irradiation; (g) photo-leakage current (*V*_GS_= −10V and *V*_DS_= 10 V) of a-IGZO TFTs in the reverse measurement as a function of photon energy of the incident light.

Figure 2a–f shows the transfer curves of the devices measured in double-scan mode without and with monochromatic light illumination. In the forward measurement, when the incident light wavelength is shorter than 460 nm, a SS degradation is distinctly observed regardless of the annealing temperature. The extent of deterioration is lower for increasing treatment temperatures, which further proves that the TFT annealed at low temperature maintains the high concentration of defects in the channel layer. The SS degradation is effectively depressed in the reverse measurement even under higher-energy light irradiation, demonstrating that the generated ionized oxygen vacancies (V_O_^+^/V_O_^2+^) are neutralized by capturing electrons [8]. However, SS degradation still can be observed in the 300 °C-annealed TFT under the irradiation with short-wavelength light (below 430 nm), indicating that light-induced defect states cannot be completely stabilized. In addition, a photo-leakage current starts to appear when the wavelength of the incident light is 460 nm. The current gradually increases with a decrease in light wavelength and annealing temperature. The influence of the incident light energy on the photo-leakage current of the devices annealed at different temperatures is displayed in Figure 2g. From using the double-scan mode, it can be concluded that the low-temperature-treated IGZO contains intrinsic defect states around the Fermi level (*E*_F_) at *V*_on_, which are added to the light-excited defect states at the same level. It is also implied that the electrical properties, including SS and off-current, directly depend on the annealing temperature regardless whether or not there is light irradiation.

**Figure 3:**
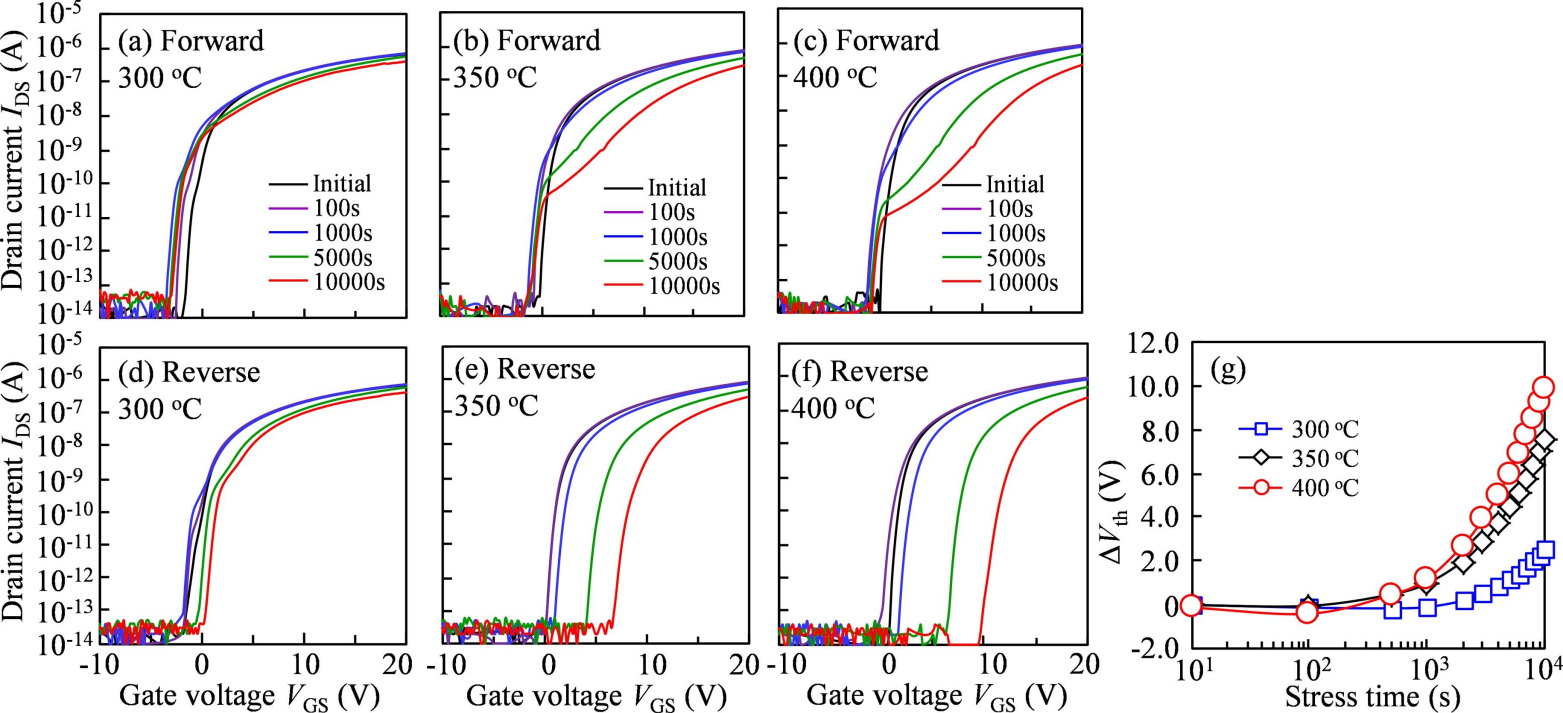
Transfer characteristics in the forward measurement for the devices annealed at (a) 300 °C, (b) 350 °C, and (c) 400 °C, and in the reverse measurement for the devices annealed at (d) 300 °C, (e) 350 °C, and (f) 400 °C as functions of stress duration under −20 V *V*_GS_ NBIS; (g) *V*_th_ values of the transfer curves of the devices as a function of NBIS duration.

The devices annealed at different temperatures were then exposed to a NBIS measurement over a period of 10^4^ s (Figure 3). In the forward measurement (Figure 3a–c), the initial transfer curve of the TFT treated at 300 °C exhibits a serious SS deterioration. The extent of SS degradation is gradually aggravated along with on-current reduction with the increasing stress duration. The photo-induced current results indicate that the incident light with the wavelength of 460 nm (ca. 2.7 eV) causes a severe SS distortion irrespective of the treatment temperature. Therefore, new defect states are generated and accumulated near *E*_F_ at *V*_on_, which originate from the high density of V_O_ near the valence band (*E*_V_). When the annealing temperature increases to 350 °C, the transfer curves exhibit outstanding initial electrical properties. However, a positive shift accompanied by the appearance of a hump and SS degradation occurs and gradually increases with increasing stress duration. The results demonstrate that the high concentration of V_O_ defects near *E*_V_ is excited to V_O_^+^/V_O_^2+^, located below the bottom of the conduction band [12]. Meanwhile, the photo-excited electron–hole pairs from the *E*_V_ are separated because of a vertical electric field (*V*_GS_ = −20 V). Consequently, the transfer curves shifted in the positive direction are attributed to generated holes trapped in the GI/IGZO interface. These phenomena are stronger when the heating temperature is further increased to 400 °C, suggesting that the more V_O_ defects exist near the *E*_V_ region. In the reverse measurement (Figure 3d–f), the initial transfer curve in the 300 °C-treated TFT still shows an acute SS distortion. The tendency of SS degradation is slightly strengthened, and there is a slightly positive shift of 2.51 V when the stress duration exceeds 1000 s. These results imply that the generated defect states cannot be completely stabilized even through scanning with high voltage. There is no hump observed in the forward scanning measurement of the 350 °C-annealed device, proving that light-induced defect states during the stress are stabilized when the measurement voltage is scanned from 20 to −10 V. Meanwhile, the transfer curves shift without SS distortion. In the case of the device annealed at 400 °C, the transfer curves further shift to 10.29 V with a stable SS (Figure 3g) that is larger than that in the case of annealing at 350 °C (7.20 V).

**Figure 4:**
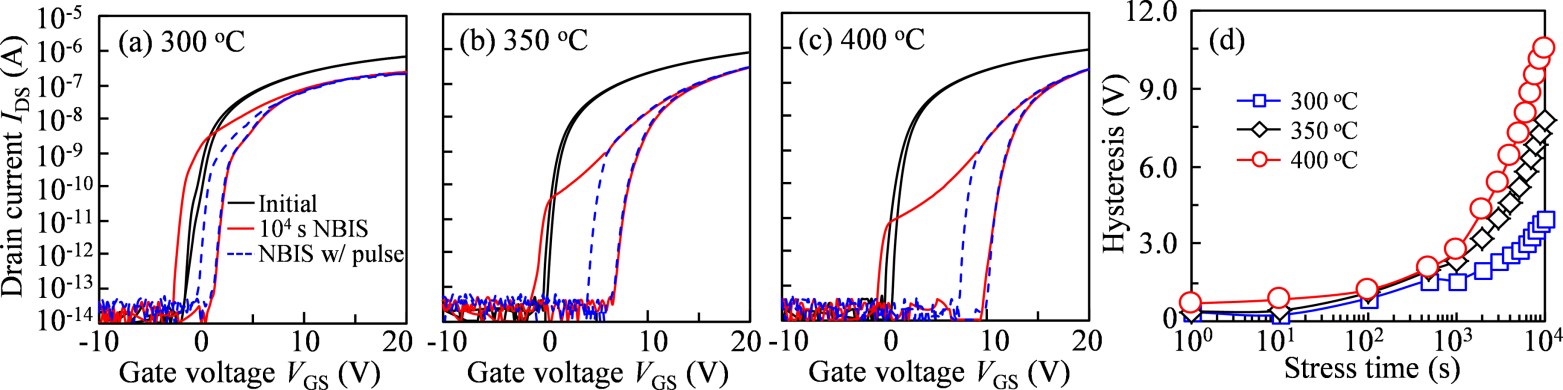
Figure 4 Transfer curves of IGZO TFTs annealed at (a) 300 °C, (b) 350 °C, and (c) 400 °C before and after 10^4^ s NBIS without and with a 10 V gate pulse; (d) hysteresis of IGZO TFTs as a function of NBIS duration.

**Figure 5:**
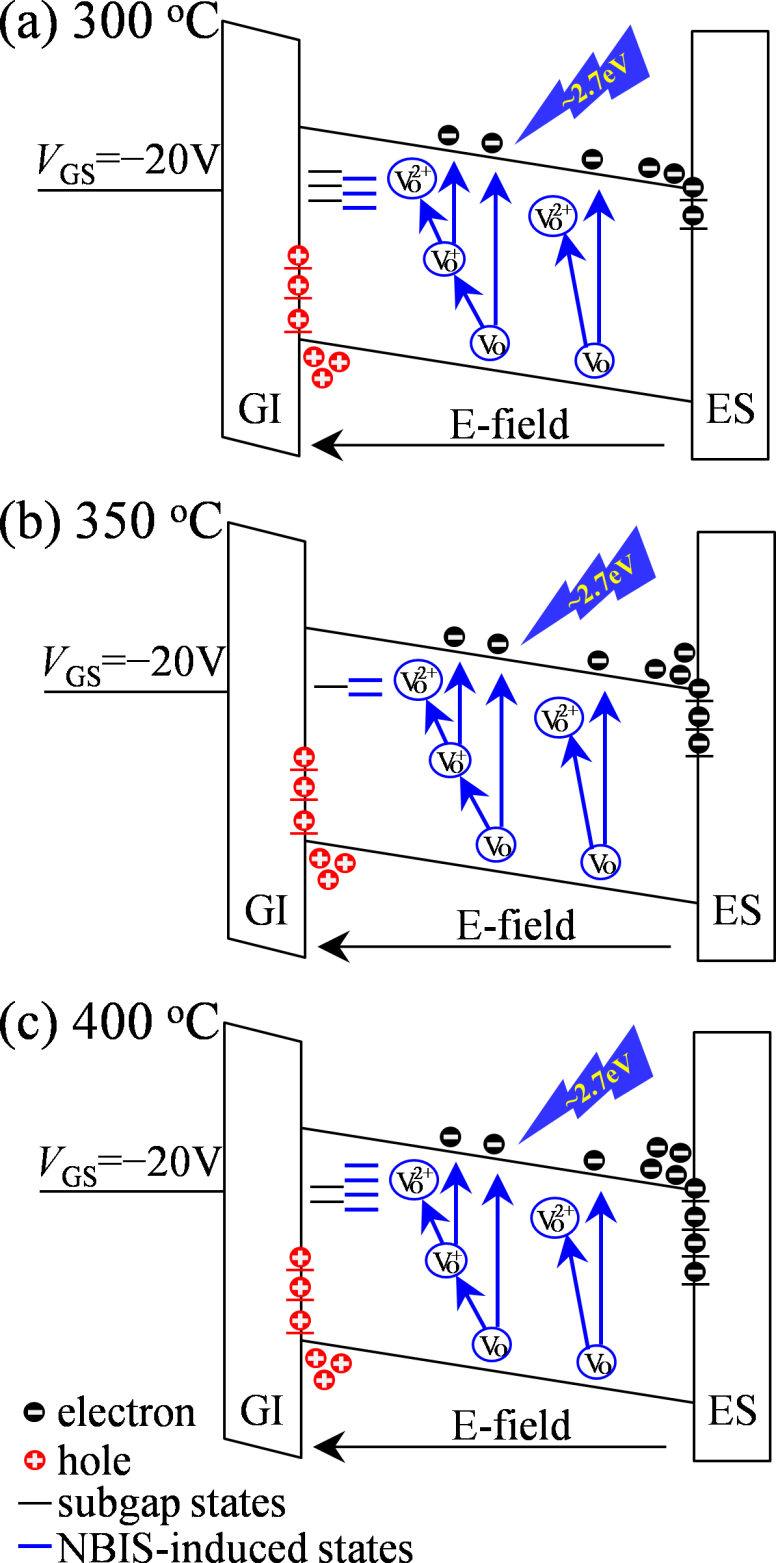
Possible degradation mechanisms of NBIS-induced instability in the devices annealed at (a) 300, (b) 350, and (c) 400 °C, respectively.

To quantitative evaluate the hysteresis caused by NBIS, a 10 V pulse with 1 ms width is exerted just after 10^4^ s stress duration and before the forward measurement. Figure 4a–c shows the transfer curves of the annealed TFTs before and after stress without and with a pulse. After a stress duration of 10^4^ s, the hysteresis values are 3.99, 7.76, and 10.55 V for the TFTs annealed at 300, 350, and 400 °C, respectively (Figure 4d). In our previous publication [13], the additional positive gate pulse can neutralize the ionized V_O_^+^/V_O_^2+^ states and release the trapped holes at the GI/IGZO interface, helping to smooth the hump induced by donor-like defect states. When the pulse is applied, the hysteresis values are 1.58, 2.75, and 2.66 V corresponding for the devices treated at 300, 350, and 400 °C, respectively. The total hysteresis induced by NBIS consists of defect-state generation in the bulk and hole trapping at the interface. The contribution of excited defect states to the hysteresis values is calculated to be 2.41, 5.01, and 7.89 V for devices annealed at 300, 350, and 400 °C, respectively. In the TFT treated at 300 °C, the incident light excites oxygen-containing defects to V_O_^+^/V_O_^2+^, which create new defect states in the IGZO. Meanwhile, electron–hole pairs are excited in the channel layer, which partly neutralize the defect states with free electrons, leaving holes near *E*_V_. Furthermore, the generated free holes and electrons are prone to be trapped because of more defect states existing in the IGZO layer (Figure 5a). Consequently, the transfer curve exhibits a severe SS degradation in the forward measurement, as well as a big SS distortion and a relatively small positive shift. In contrast, for the device annealed at 350 °C, the created donor-like states in the forward measurement are completely stabilized in the reverse scanning, and photo-induced carriers are driven by the electric field and drift to the interfaces (Figure 5b). In virtue of the relative high quality of the IGZO bulk as well as its adjacent interfaces, the transfer curves show only a small hump and a small gap-shift without SS distortion in the forward and reverse scanning. When the TFT is annealed at 400 °C, the attenuation of the electrical properties is equivalent to that of the 350 °C sample. However, due to the lesser quality of the IGZO layer and its adjacent interfaces, the transfer curves exhibit a big hump in the forward scan and a long-range shift in the reverse scan. Furthermore, when the double-scan mode and a positive gate pulse are combined, as schematically illustrated in Figure 5c, a higher number of defect states is excited in the semiconductor bulk and an identical amount of holes is trapped at the interfaces in comparison with the results in the TFT annealed at 350 °C.

Based on the abovementioned results, it is suggested that the annealing process provides the possibility to regulate the qualities of the channel layer as well as its adjacent interfaces, thereby controlling the initial electrical characteristics and bias-stress stabilities of the devices. In this study, the TFT annealed at 350 °C exhibits an outstanding initial performance, but its long-term stability has been deteriorated to some degree under the high-energy light irradiation. Therefore, to further improve the electrical properties and bias-stress stabilities, especially against NBIS, the semiconductor active layer with appropriate stoichiometry during the fabrication should be controlled. Also, the interface engineering in high-performance TFTs, including surface treatment and buffer layer introduction [14–15], should be carefully considered.

## Conclusion

The influence of annealing temperature on the initial electrical characteristics and photo-induced instabilities in a-IGZO TFTs is explored. The electrical parameters reveal that annealing at low and high temperatures leads to the creation of high densities of defects in the IGZO bulk and its adjacent interfaces, respectively. The high-energy incident light excites the defect generation and the leakage current increases in the devices regardless of annealing temperature. Using the double-scan mode and a positive gate pulse, the NBIS-caused hysteresis for the TFTs annealed at different temperatures is quantitatively clarified.
